# Clinical experience and outcomes of post chemotherapy midline extraperitoneal approach to retroperitoneal lymph node dissection

**DOI:** 10.1186/s12894-025-01853-0

**Published:** 2025-07-14

**Authors:** Basil Razi, James Kovacic, Ankur Dhar, Andrew Shepherd, Venu Chalasani, Matthew Winter

**Affiliations:** 1 Department of Urology, Royal North Shore Hospital, St Leonards, NSW Australia; 2 North Shore Urology Research Group (NSURG), Royal North Shore Hospital, St Leonards, NSW Australia; 3 University of Sydney, Sydney, NSW Australia; 4 Adelaide Medical School, University of Adelaide, Adelaide, SA Australia

**Keywords:** Extraperitoneal, Oncology, Open surgery, RPLND, Testicular Cancer

## Abstract

**Background:**

To detail the outcomes of an open midline extraperitoneal (midline EP) approach to post-chemotherapy retroperitoneal lymph node dissection (PC-RPLND) for metastatic testicular cancer.

**Methods:**

We analysed our prospectively maintained operative database from April 2020 to February 2023 for cases of midline EP approach to PC-RPLND, identifying a total of 11 patients across two hospitals in Sydney, Australia. Demographic and perioperative data was obtained from electronic medical records, including preoperative factors such as cancer staging and preoperative treatment.

**Results:**

Eleven patients were included in this study. The median age was 37 years with a median ASA grade of 3. There were a total of six left-sided and five right-sided cases. A modified template was used in eight cases, and a bilateral template was used in three. Tumour staging ranged from Stage IIA– IIIB, with a median maximal retroperitoneal tumour size post chemotherapy of 4.2 cm. Preoperative histology identified 4 cases of seminoma and 7 cases of nonseminomatous germ cell tumours (NSGCT). The median length of the procedure was 300 min, blood loss was 300mL, length of stay was 5 days, and post-operative days until bowel opening was 2 days. The median lymph node yield was 18, with active malignancy identified in five cases. There were four early complications and no late complications. 91% of the patients had preserved ejaculatory function.

**Conclusions:**

The open midline EP approach to PC-RPLND has demonstrated acceptable perioperative outcomes compared to other open surgical approaches, enabling surgeons to complete complex cases. Therefore, the midline EP approach should be considered when performing PC-RPLND.

**Supplementary Information:**

The online version contains supplementary material available at 10.1186/s12894-025-01853-0.

## Introduction

Testicular cancer is the second most common malignancy in young men, representing 1% of adult male neoplasms and 5% of all urological cancers [1, 2]. Retroperitoneal lymph node dissection (RPLND) plays an important role in the management of testicular cancer, both as a staging and therapeutic procedure for seminomatous (SGCT) and nonseminomatous germ cell tumours (NSGCT) [3]. RPLND is a technically demanding procedure, with an estimated overall complication rate of 20–35% and a mortality of 1% [4, 5]. There are two traditional methods of accessing the retroperitoneum, either extraperitoneal (EP) accessed via the flank or a midline transperitoneal (TP) approach [6, 7]. In our geographical region (Australasia), RPLND is most commonly used as a therapeutic procedure for post-chemotherapy residual retroperitoneal masses in metastatic testicular cancer.

An alternative technique that is seldom used is the open midline EP approach, which allows for excellent exposure of retroperitoneal structures and exposure of the contralateral side if required, while avoiding the morbidity associated with a flank incision or transperitoneal access. Studies have demonstrated significantly improved patient outcomes and shorter hospital length of stay with an extraperitoneal approach [3, 8, 9], contributing to the lower rates of post-operative ileus that have historically prolonged the length of stay for TP-RPLND.

Robot assisted transperitoneal RPLND is an increasingly common approach, however it has a significant learning curve, added financial costs and robotic units are not widely available in Australia [10, 11]. In comparison, the open midline EP approach has been safely used within our centre for surgery of retroperitoneal pathology such as in RPLND, radical nephrectomy with IVC thrombectomy, partial nephrectomy, and resection of pararenal sarcoma. Recent reports have demonstrated an equivalent oncological lymph nodal yield with robotic RPLND compared to open [12–14]. In the literature, the midline EP approach has been used across surgical specialties. Vascular surgeons have described the approach for open abdominal aortic aneurysm repairs [15–17], and general surgeons have described its use for obturator hernia repair [18] and retroperitoneal abscess management [19, 20]. There is limited published evidence on the use of a midline EP approach for RPLNDs, however a study by The University of Southern California has identified improved outcomes in post-chemotherapy (PC) RPLND utilising the midline EP approach [8, 9].

This report represents the first Australian series detailing the open midline EP approach to PC-RPLND for metastatic testicular cancer and aims to serve as an example of how this surgical approach can be used in complex surgery to minimise patient morbidity.

## Methods

### Data accumulation and patient selection

Consecutive patients were selected for inclusion if they had undergone an open midline EP approach to RPLND following systemic treatment for metastatic testicular cancer performed by a urologist associated with the Northern Sydney or Nepean Blue Mountain local health districts in Sydney, Australia. Eleven patients across two hospitals were identified from our prospectively maintained operative database between April 2020 and February. Demographic, pre-operative, and post-operative outcome data were collected through retrospective review of electronic medical records.

### Pre-operative patient selection

Patients were selected for operative management if they had interval growth on surveillance imaging of a retroperitoneal mass following systemic therapy for metastatic testicular cancer, or if they were symptomatic of disease by way of persistent pain or ureteric obstruction. Each patient was discussed at a multidisciplinary oncology meeting prior to proceeding with operative intervention.

### Surgical approach

**Fig. 1:**
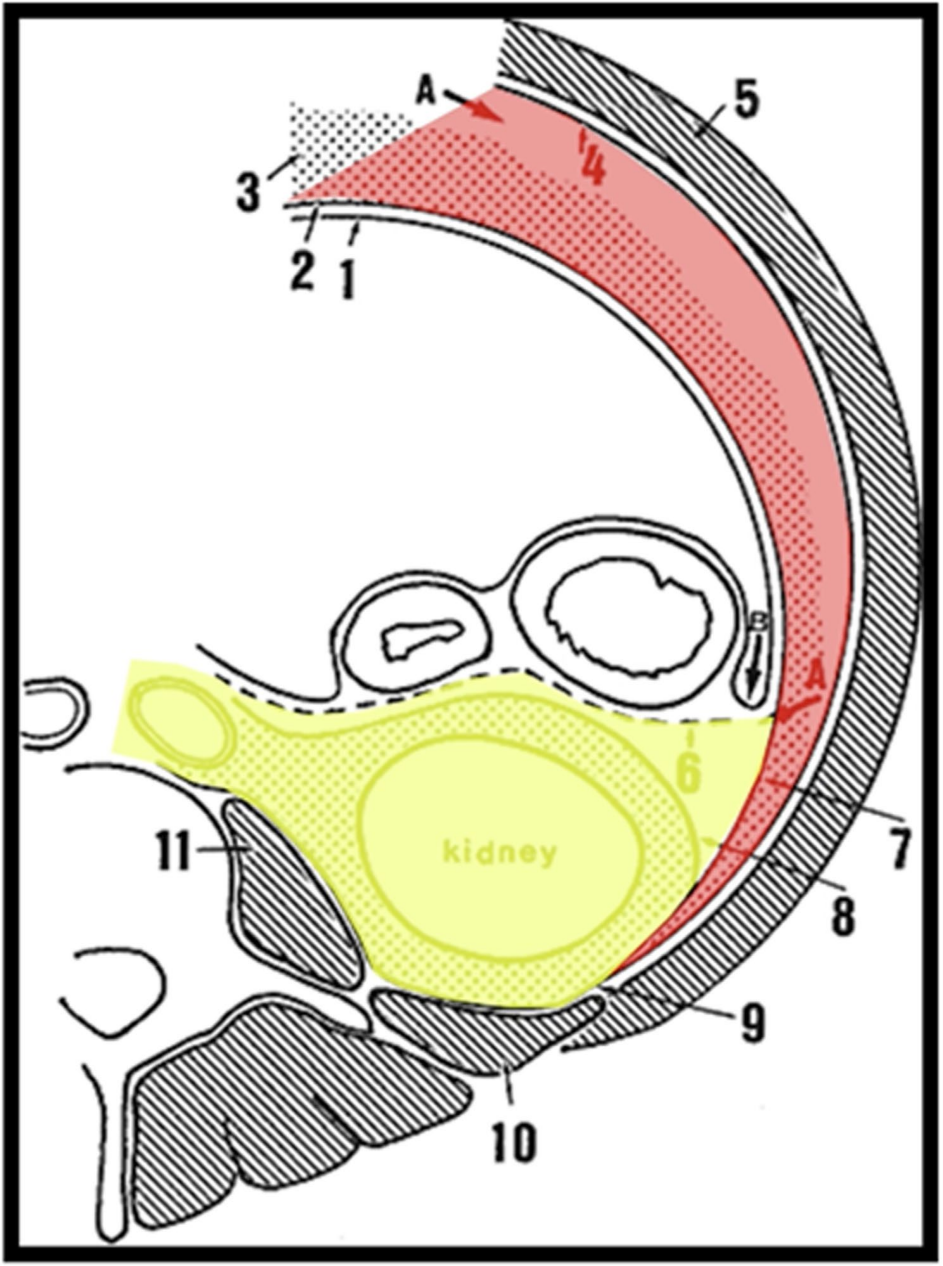
Abdominal schematic. Red shaded region: Pre-peritoneal space. Yellow shaded region: Retroperitoneum. 1) Visceral peritoneum. 2) Parietal peritoneum. 3) Pre-peritoneal fat. 4) Tr*a*nsversalis fascia. 5) Transversus abdominus muscle. 6) Fusion fascia. 7) Lateroconal fascia. 8) Gerota’s fascia, anterior lamina. 9) Gerota’s fascia, posterior lamina. 10) Quadratuslumborum muscle. 11) Psoas muscle

Our series identified midline EP RPLND cases from a single urological surgeon, fellowship trained in open and robotic oncological urological surgery. The described approach can be utilised for bilateral or modified template dissection of retroperitoneal structures. Please see Fig. 1 for the anatomical schematic and Video Supplement 1.

**Fig. 2:**
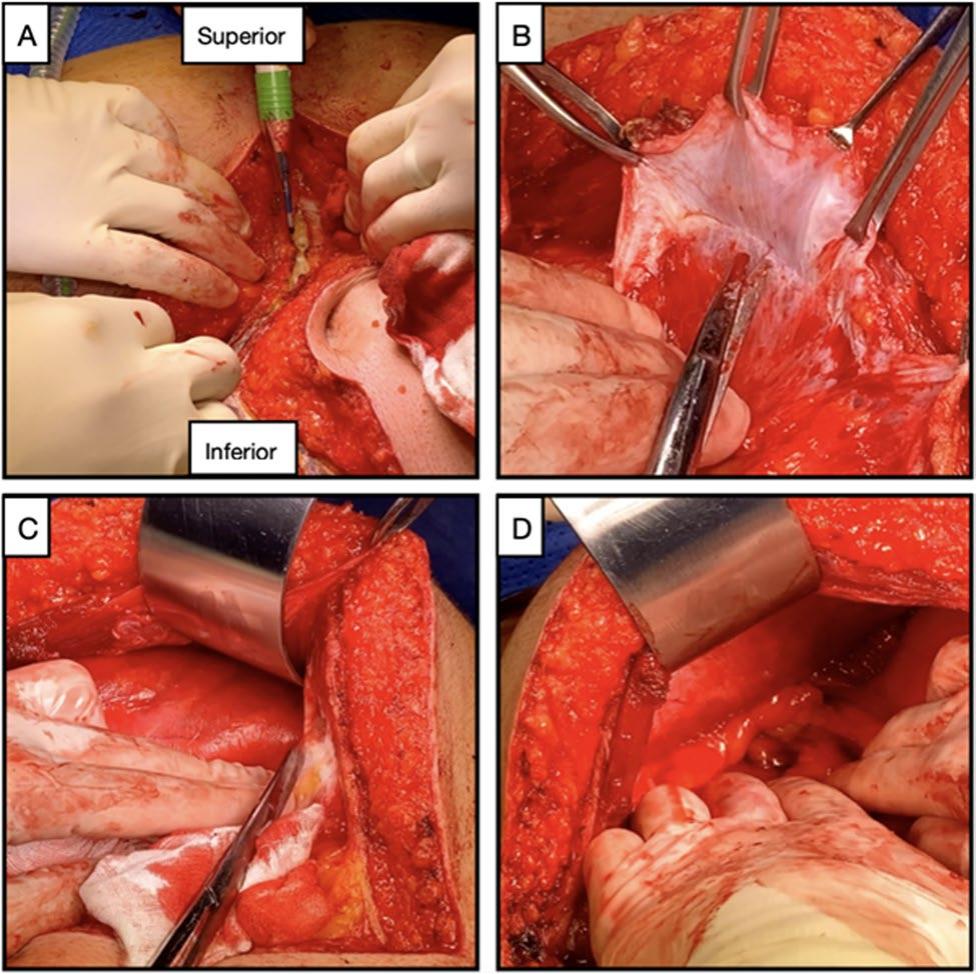
**A** Midline laparotomy wound, diathermy incision through rectus sheath. **B** Rectus sheath retracted superiorly. Peritoneum inferiorly. **C** Lateral advancement of extraperitoneal plane. **D** Identification of retroperitoneal structures

The patient was positioned supine with routine preparation and a square-drape. A midline incision was made 20–25 cm in length extending from infra-xiphoid to infra-umbilicus, and the incision was extended as required. Dissection was then carried to the rectus sheath, which was carefully entered with cranial and caudal extension. The transversalis fascia was divided, and the preperitoneal space developed without breaching the parietal peritoneum. Using a combination of blunt and sharp dissection, the preperitoneal plane was developed laterally while evenly spreading tension on the peritoneum using sponges. This is both a critical procedural step to enable exposure of the retroperitoneum, but it can also be time-consuming and complicated by a peritoneal breach. If a peritoneal breach occurs inadvertently, it should be immediately repaired using a 3 − 0 absorbable suture to prevent widening of the hole or spillage of the peritoneal contents. See Fig. 2 for examples of initial dissection.

**Fig. 3:**
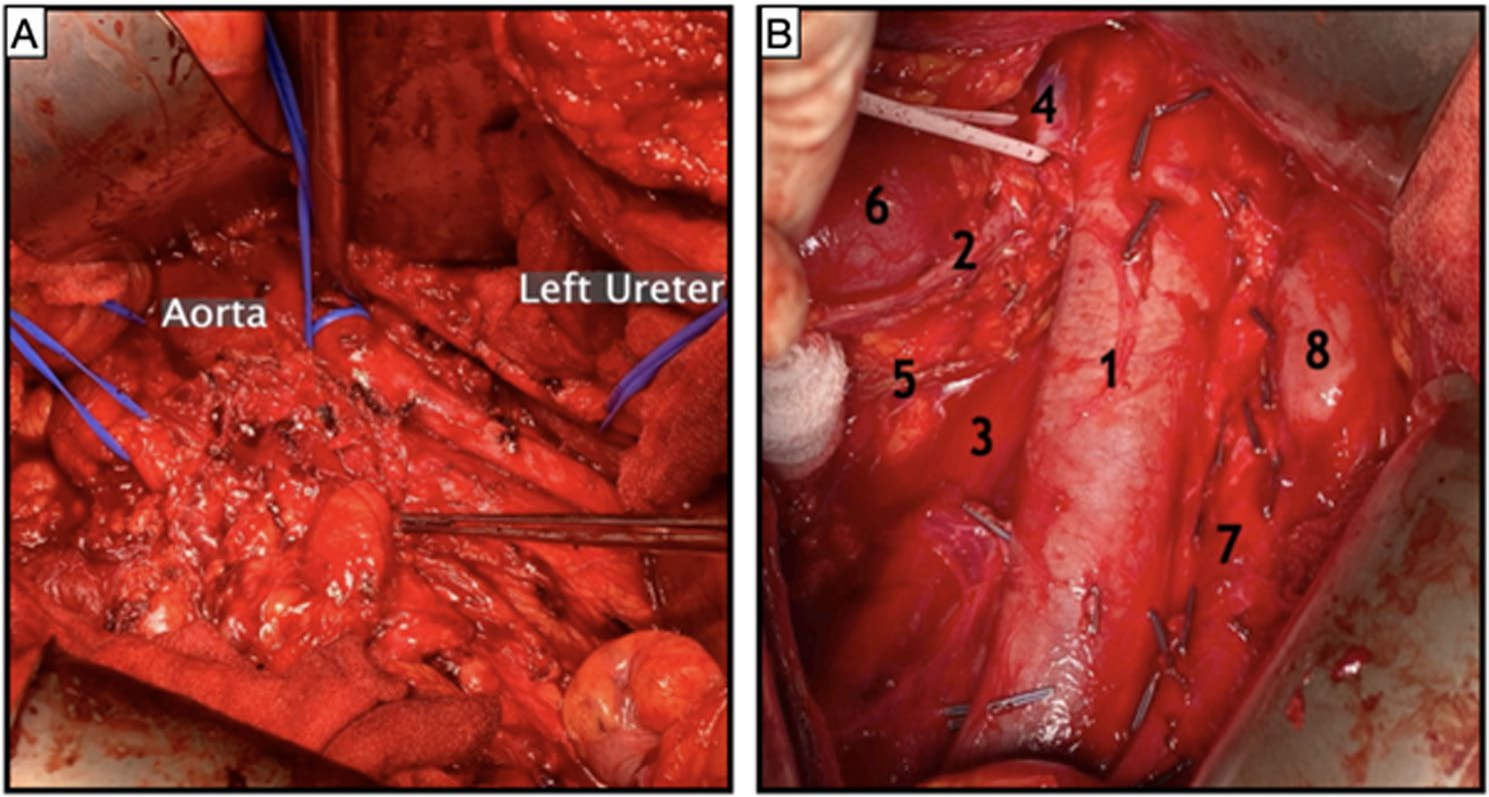
**A** Retroperitoneum prior to lymph node dissection. Right ureter, abdominal aorta, and left ureter with vessel loops. Forceps point at large soft tissue mass. **B** 1. Inferior vena cava. 2. Right ureter. 3. Right psoas muscle. 4. Right renal vein. 5. Right gonadal vessels. 6. Right kidney. 7. Aorta. 8. Duodenum

Upon reaching the lateroconal fascia, the peritoneal sac was retracted superomedially to expose the retroperitoneum. We used an Omnitract (Integra LifeSciences, Princeton, New Jersey, USA) fixed body wall retractor to maintain exposure, with use of handheld abdominal wall retractors, as required, to facilitate finer points of dissection. Wet sponges were placed beneath the body wall retractors. We performed nerve sparing where possible, sparing lumbar arteries and lumbar veins. The IMA was ligated to allow more complete visualisation of the left retroperitoneum which was of particular importance for bilateral cases. Following exposure, RPLND is performed in a routine manner using either a full bilateral template for cases of right-sided testicular tumours or a modified template for left-sided testicular tumours, depending on preoperative imaging and operative findings. Our series also included two cases of modified (unilateral) RPLND for recurrence of retroperitoneal masses following initial RPLND 15 and 25 years prior. See Fig. 3 as an example.

**Fig. 4:**
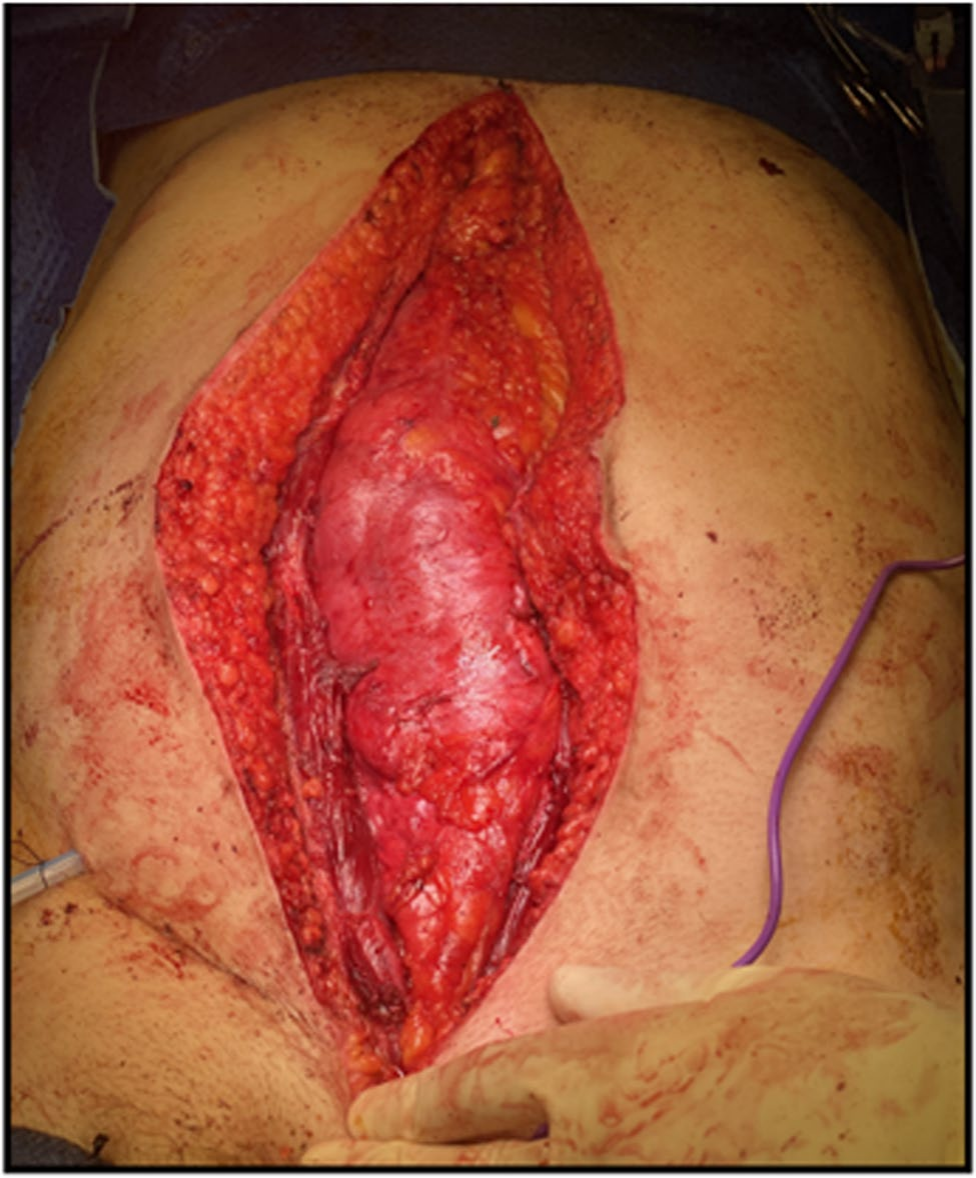
Peritoneum remains intact at case conclusion

Ancillary procedures such as ipsilateral nephrectomy can be performed using the same approach. A 15Fr Blake drain was placed in the retroperitoneal space, and closure of the rectus fascia was completed with a 0-polydioxanone suture, with subcutaneous fat and skin closed according to surgeon’s preference. As shown in Fig. 4, the peritoneum remained intact throughout the case.

Post-operative analgesia was commenced using patient-controlled analgesia (PCA) with routine DVT prophylaxis commencing the night of surgery. The patient was started on a low-fat diet immediately after surgery to reduce the risk of chyle collection [21]. The drain is removed once the patient is mobilising, producing a volume of less than 50mL per day, which can be facilitated as an outpatient if required.

### Complications

Intra-operative complications were graded according to the Oslo Classification system [22]. Early complications were defined as occurring within 30-days of surgery, and severity was assessed using the Clavien-Dindo classification [23]. Late complications were deemed to have occurred if beyond 30-days after surgery.

## Results

### Patient characteristics

A retrospective review identified 11 consecutive patients who underwent an open midline EP approach to RPLND for metastatic testicular cancer performed by a single surgeon (Table 1). The median patient age was 37 years (range 26–51 years). All patients had an ASA score of 3. All but one patient (Stage IIIB undergoing upfront systemic therapy with elevated tumour markers and an avid left testicle on FDG-PET) had undergone radical inguinal orchidectomy in the past (five left-sided and five right-sided). A bilateral template was used in 3 cases, and a modified (unilateral) template was used in the remaining 8 cases. Preoperative histopathology included 4 cases of seminoma and 7 cases of NSGCT. Each patient received bleomycin, etoposide, and cisplatin (BEP) therapy with a median of three cycles (range 2–4 cycles). TNM staging prior to RPLND included 1 patient with Stage IIA, 4 with Stage IIB, 4 with Stage IIC, 1 with Stage IIIA and 1 patient with Stage IIIB disease. The median residual retroperitoneal tumour size of 4.2 cm (interquartile range (IQR): 1.8–5). Preoperative hydronephrosis was managed with a ureteric stent in three cases prior to proceeding with RPLND.

**Table 1:** Patient demographics

Patients (*n*)	11	
Age, median (years)	37 (range 26–51)	
ASA	III (*n* = 11)	
Initial histology	Seminoma	4
	Non-seminoma	7
Pre-operative staging	Stage IIA	1
	Stage IIB	4
	Stage IIC	4
	Stage IIIa	1
	Stage IIIb	1
Initial tumour markers	Normal	7
	Elevated	4
Pre-RPLND procedures	Radical Orchidectomy	10
	Ureteric stent	3
	Prior RPLND	2

### Intra-operative complications

Two patients were identified as having Oslo classification grade 2 intra-operative complications. In one case, an inadvertent injury occurred during dissection of the right common iliac artery (as demonstrated in Video Supplement 1) while grasping the artery with Debakey forceps. The proposed reason for the injury was entering the arterial adventitia during dissection of the fibrotic mass encasing the structure, subsequently leading to arterial wall fragility. Owing to poor tissue quality, the injury could not be closed primarily, but instead was repaired with the assistance of a vascular surgeon using a polytetrafluoroethylene (PTFE) graft to good effect. This event was an outlier that led to a significant blood loss of 2600mL during what became the longest case of our series at 615 min. Another intra-operative vascular injury occurred in the left renal vein, which was oversewn with 5 − 0 Prolene primarily and resulted in an additional 200mL of blood loss. This injury occurred during the dissection of a 5 cm adherent mass from the abdominal aorta and left renal vein. No other intra-operative complications occurred in the series.

### Peri-operative results

The median length of the procedure was 300 min (IQR: 280–345), median operative blood loss was 300mL (IQR: 100–875), and length of stay was 5 days (range 3–10 days). The median number of post-operative days until the opening of the bowels was 2 (range 1–3 days).

Post-operative histology revealed a median nodal count of 18 lymph nodes (range 1–39). The median nodal count for a bilateral template was 33 lymph nodes (IQR: 20–39) and seven nodes (IQR: 2.5–17.5) for a modified unilateral template. Of the 11 cases, five demonstrated viable GCT, three showed necrosis or fibrosis and three contained a teratoma.

A subgroup analysis comparing seminoma and NSGCT revealed notable differences. Seminoma patients had larger retroperitoneal masses (median 6.85 cm vs. 3.6 cm), however yielded a lower number of lymph nodes (median 11.5 vs. 17). The median operative time was longer for seminoma patients (322 vs. 283 min). Regarding ancillary procedures, nephrectomies were performed in all four seminoma patients, only 1 NSGCT patient also underwent an orchidectomy.

### Early (< 30-day) complications

In total, four early complications occurred in four patients. There were two cases of chyle leaks (one managed conservatively and the other requiring radiological drain insertion), wound infection, and lymphocele requiring radiological drainage. A single readmission was encountered in one of the above patients who presented with a chylous collection. Perioperative results are shown in Table 2.

**Table 2:** Peri-operative information and results

Operative duration, median (min)	300 (IQR: 280–345)	
Blood loss, median (mL)	300 (IQR: 100–875)	
ICU admission (n=)	7	
Length of stay, median (days)	5 (IQR: 5–7)	
Time to bowels open, median (days)	2 (range 1–3)	
Intraoperative complication, Oslo classification (n=)	I	0
	II	2
	III	0
Early complication (< 30-days), Clavien-Dindo classification, (n=)	I	2 (wound infection; chyle leak)
	II	0
	IIIa	2 (chyle leak; lymphocele)
	IIIb	0
	IV	0
	V	0
RPLND template	Modified	8
	Bilateral	3
Nodal count, median	Modified	7 (IQR: 2.5–17.5)
	Bilateral	33 (IQR: 20–39)
Ancillary procedures	Nephrectomy	4
	Orchidectomy	1

### Late (> 30-day) complications

No late complications occurred during the study period.

### Functional outcomes

Antegrade ejaculation was maintained in 91% (10/11) of the patients, based on patient self-reports obtained during routine postoperative clinic follow-up. Anejaculation was encountered in a single post-operative patient with high-risk histopathology of extranodal spread into fat, managed with adjuvant radiotherapy.

### Ancillary procedures

Four patients underwent ipsilateral nephrectomies. Three were performed secondary to fibrotic encasement of the ureter, with severe hydronephrosis and compromised renal function. One patient was identified as having extensive renal vein thrombosis secondary to a dense desmoplastic reaction to chemotherapy.

One patient underwent a planned cavotomy secondary to a large fibrotic mass that was adherent to the anterior inferior vena cava. Cavotomy was repaired with the assistance of a vascular surgeon using a bovine patch graft to good effect.

A single patient in our study underwent left radical orchidectomy during RPLND. This patient presented to the hospital with de novo metastatic Stage IIIB disease and underwent upfront BEP therapy prior to the completion of RPLND and orchidectomy. Orchidectomy was performed using the same midline EP laparotomy incision, without extension of the wound toward the groin.

### Follow-up

No patients were lost to follow up. The overall median follow-up after RPLND was 600 days (range 60–1095 days). Oncological surveillance was performed according to EAU guidelines, with one case identified as having systemic and local failure 6-months post RPLND treated with adjuvant radiotherapy. This particular case included a 55 mm conglomerate of lymph nodes, 90% of which were viable seminomas with extranodal extension but negative surgical margins. Recurrence was identified in the left para-aortic node and left supraclavicular node. The remaining patients were recurrence-free.

## Discussion

In this retrospective series, we identified low rates of post-operative complications without compromising oncological control via the open midline EP approach to PC-RPLND. This procedure and clinical scenario are notoriously challenging for surgeons because of limited exposure and a dense desmoplastic reaction secondary to systemic therapy. We believe that the extraperitoneal approach is beneficial for exposure and dissection, and this assists in minimising the risk of post-operative complications such as ileus and secondary bleeds. Chyle leaks in the extraperitoneal space are more likely to become symptomatic due to the confined space, which may account for the higher leak rate of 18% observed in this series, compared to the 5–10% reported in the open RPLND literature [24, 25]. In open RPLND, CT scans are typically performed only for symptomatic patients. The midline EP approach was associated with an early return to bowel function while maintaining equivalent oncological outcomes; however, we acknowledge that there are limitations in this series.

There are several reasons why the open midline EP method is our preferred approach to PC-RPLND compared to traditional access via either a midline TP or extraperitoneal flank incision. In comparison to the TP approach, midline EP surgery has the benefit of reducing operative intra-peritoneal complications, while minimising post-operative complications such as ileus and adhesions. In our series, we did not observe any intra-abdominal injuries or post-operative ileus. This stands in contrast with the literature, which has identified significant risk of post-operative ileus and intra-abdominal adhesions following a TP approach to surgery [4, 26].

The EP flank approach, in comparison, shares similarities with the midline EP approach; however, it has several drawbacks. The EP flank approach carries a higher risk of pneumothorax, subcostal nerve injury with resultant loss of sensation, and post-operative pain [27]. Another significant factor in choosing the midline EP approach is bilateral access to the retroperitoneum, which is not feasible via a single-flank incision [27].

Despite the small sample size, our study demonstrated similar findings with those previously reported by the University of Southern California group, which represents the only other investigations into the open midline EP approach to PC-RPLND [8, 9]. Our study supports the finding that this technique contributes to a shorter length of stay, with our median length of stay of 5 days being less than that of alternative open approaches identified in large-scale retrospective reviews performed by Macleod and Notarfrancesco, who identified 9- and 7-day LOS respectively [22, 23]. The shorter length of hospital stay in our series was thought to be partly explained by the low incidence of ileus.

The open midline EP approach, although with limited evidence in the literature, seems to have improved perioperative outcomes compared to alternative open approaches. However, surgery in general is shifting toward a minimally invasive approach, and robot-assisted RPLND has become more feasible in high-volume centres than prior attempts at laparoscopic RPLND [11]. A recently published systematic review by Garg et al. demonstrated equivalent outcomes with open PC-RPLND performed by expert surgeons using the robotic approach [12]. Garg identified 188 patients across the literature who underwent robot-assisted PC-RPLND, with median ranges including operative time 134–550 min, blood loss 43–2300mL, median length of stay 1–8 days, and an open conversation rate of 9%. The risks of major complications were similar between the robotic and open approaches. However, there has been discussion in the literature regarding the decreased oncological safety and completion of lymph node dissection when using a minimally invasive approach secondary to malignant seeding and changes in tumour biology secondary to pneumoperitoneum [28]. Within the Australian surgical landscape, there are logistical issues as to why routine robotic surgery is not feasible, including limited public access to robotic surgical systems and a lack of high-volume centres that may be found abroad.

The authorship group acknowledges that this study has several limitations; however, it remains one of the few series to assess the open midline approach to PC-RPLND. Key limitations include its retrospective design, absence of internal comparators (either alternative open approach or robot-assisted), single-surgeon data, and a small volume of cases. In this series, the surgeon was already well experienced in RPLND techniques, however the influence of a learning curve specific to the for a midline EP approach cannot be excluded. This may limit the broader applicability of this approach, particularly given the technical complexity of PC-RPLND. One outcome of our study that stands out in comparison with other series is the high rate of ancillary nephrectomy. Each patient who underwent nephrectomy in our series had hydronephrosis secondary to either malignant obstruction or dense fibrosis encasing the ureter. Preoperative renal split-function was assessed for each patient, and discussion was performed with patients to ensure informed consent was gained, given that the risk of ureteric reconstruction in a densely fibrotic surgical field would be unlikely to yield positive outcomes.

Our study represents the first Australian series to assess the safety of the open midline EP approach to PC-RPLND, and while a small case volume, it has demonstrated acceptable intraoperative and perioperative risk profiles compared with alternative approaches to surgery.

## Conclusions

PC-RPLND remains a necessary treatment option for patients with metastatic testicular cancer; however, it has historically had a high surgical morbidity. While the role of minimally invasive surgery for this indication continues to evolve, it should not yet be considered a standard practice unless performed by experienced urologists with familiarity of RPLND or outside of tertiary referral centres. The open midline EP approach has demonstrated favourable perioperative outcomes and should be considered as an alternative approach when undertaking PC-RPLND.

## Supplementary Information

Supplementary Material 1.

## Data Availability

Data is provided within the manuscript and supplementary information files.

## References

[1] Park JS, Kim J, Elghiaty A, Ham WS. Recent global trends in testicular cancer incidence and mortality. Med (Bmore). 2018;97(37):e12390. 10.1097/MD.0000000000012390 PMC6155960 30213007

[2] Siegel RL, Miller KD, Wagle NS, Jemal A. Cancer statistics. CA Cancer J Clin. 2023;73(1):17–48. 36633525 10.3322/caac.21763

[3] Kim P, Syan-Bhanvadia S, Djaladat H, Faber K, Tadros NN, Nichols C, et al. Midline extraperitoneal approach for retroperitoneal lymph node dissection for testicular germ cell tumor. Urology. 2012;80(4):941–5. 22951007 10.1016/j.urology.2012.07.006

[4] Baniel J, Sella A. Complications of retroperitoneal lymph node dissection in testicular cancer: primary and post-chemotherapy. Semin Surg Oncol. 1999;17(4):263–7. 10588855 10.1002/(sici)1098-2388(199912)17:4<263::aid-ssu7>3.0.co;2-6

[5] Williams SB, McDermott DW, Winston D, Bahnson E, Berry AM, Steele GS, et al. Morbidity of open retroperitoneal lymph node dissection for testicular cancer: contemporary perioperative data. BJU Int. 2010;105(7):918–21. 19747353 10.1111/j.1464-410X.2009.08888.x

[6] Tessler AN, Yuvienco F, Farcon E. Paramedian extraperitoneal incision for total nephroureterectomy. Urology. 1975;05(3):397–8. 1119005 10.1016/0090-4295(75)90166-1

[7] Ulm AH. Transabdominal extraperitoneal rectus-retracting incision for surgery of the kidney and ureter. J Urol. 1969;101(6):797–800. 5771245 10.1016/s0022-5347(17)62428-5

[8] Syan-Bhanvadia S, Bazargani ST, Clifford TG, Cai J, Miranda G, Daneshmand S. Midline extraperitoneal approach to retroperitoneal lymph node dissection in testicular cancer: minimizing surgical morbidity. Eur Urol. 2017;72(5):814–20. 28325537 10.1016/j.eururo.2017.02.024

[9] Alsyouf M, Ghoreifi A, Ashrafi A, Ladi-Seyedian SS, Ahmadi H, Burg M, et al. Eleven-year experience with midline extraperitoneal retroperitoneal lymph node dissection for germ cell tumors. J Urol. 2025;213(1):60–70. 39269913 10.1097/JU.0000000000004246 PMC12708023

[10] Calaway AC, Einhorn LH, Masterson TA, Foster RS, Cary C. Adverse surgical outcomes associated with robotic retroperitoneal lymph node dissection among patients with testicular cancer. Eur Urol. 2019;76(5):607–9. 31174891 10.1016/j.eururo.2019.05.031

[11] Ray S, Pierorazio PM, Allaf ME. Primary and post-chemotherapy robotic retroperitoneal lymph node dissection for testicular cancer: a review. Transl Androl Urol. 2020;9(2):949–58. 32420211 10.21037/tau.2020.02.09 PMC7214990

[12] Garg H, Mansour AM, Psutka SP, Kim SP, Porter J, Gaspard CS, et al. Robot-assisted retroperitoneal lymph node dissection: a systematic review of perioperative outcomes. BJU Int. 2023;132(1):9–30. 36754376 10.1111/bju.15986

[13] Chavarriaga J, Atenafu EG, Mousa A, Langleben C, Anson-Cartwright L, Jewett M, et al. Propensity-matched analysis of open versus robotic primary retroperitoneal lymph node dissection for clinical stage ii testicular cancer. Eur Urol Oncol. 2024;7(5):1034–41. 38278693 10.1016/j.euo.2024.01.006

[14] Ghoreifi A, Sheybaee Moghaddam F, Mitra AP, Khanna A, Singh A, Chavarriaga J, et al. Oncological outcomes following robotic postchemotherapy retroperitoneal lymph node dissection for testicular cancer: a worldwide multicenter study. Eur Urol Focus. 2024;S2405-4569(24):00241–4. 10.1016/j.euf.2024.11.001. 10.1016/j.euf.2024.11.001 39551650

[15] Igari T, Hoshino S, Iwaya F, Satokawa H, Midorikawa H, Takase S, et al. Results of 256 consecutive abdominal aortic aneurysm repairs using extraperitoneal approach. Cardiovasc Surg. 2001;9(3):249–53. 11336848 10.1177/096721090100900307

[16] Quick CR, Chan CL, Sonoda LI, Hart AJ. Midline extraperitoneal approach for elective abdominal aortic aneurysm repair. Eur J Vasc Endovasc Surg. 1997;14(1):63–8. 9290562 10.1016/s1078-5884(97)80227-4

[17] Rob C. Extraperitoneal approach to the abdominal aorta. Surgery. 1963;53:87–9. 13974200

[18] Otsuki Y, Konn H, Takeda K, Koike M. Midline extraperitoneal approach for obturator hernia repair. Keio J Med. 2018;67(4):67–71. 29540635 10.2302/kjm.2017-0014-OA

[19] Aydin O, Pehlivanli F, Karaca G, Aydin G, Ozler I, Daphan C. Suprapubic midline extraperitoneal approach for widespread retroperitoneal abscess originating from anorectal abscess. Am Surg. 2018;84(1):17–9. 29642980

[20] Okuda K, Oshima Y, Saito K, Uesaka T, Terasaki Y, Kasai H, et al. Midline extraperitoneal approach for bilateral widespread retroperitoneal abscess originating from anorectal infection. Int J Surg Case Rep. 2016;19:4–7. 26701843 10.1016/j.ijscr.2015.12.001 PMC4756073

[21] Wang K, Xiao J, Li L, Li X, Yang Y, Liu Z, et al. The application of a medium-chain fatty diet and enteral nutrition in post-operative chylous leakage: analysis of 63 patients. Front Nutr. 2023;10:1128864. 37545584 10.3389/fnut.2023.1128864 PMC10399236

[22] Notarfrancesco M, Fankhauser CD, Lorch A, Ardizzone D, Helnwein S, Hoch D, et al. Perioperative complications and oncological outcomes of post-chemotherapy retroperitoneal lymph node dissection in patients with germ cell cancer at two high-volume university centres in Switzerland - a retrospective chart review. Swiss Med Wkly. 2023;153:40053. 37080191 10.57187/smw.2023.40053

[23] Macleod LC, Rajanahally S, Nayak JG, Parent BA, Ramos JD, Schade GR, et al. Characterizing the morbidity of postchemotherapy retroperitoneal lymph node dissection for testis cancer in a national cohort of privately insured patients. Urology. 2016;91:70–6. 26802801 10.1016/j.urology.2016.01.010 PMC5679272

[24] Qian Z, D’Andrea VD, Xiao B, Kielhofner J, Yim K, Stone BV, et al. Contemporary perioperative outcomes of open retroperitoneal lymph node dissection for testicular cancer. JU Open Plus. 2024;2(3):e00013. 10.1097/JU9.0000000000000114.

[25] Shishido T, Okegawa T, Hayashi K, Masuda K, Taguchi S, Nakamura Y, et al. Laparoscopic retroperitoneal lymph node dissection versus open retroperitoneal lymph node dissection for testicular cancer: a comparison of clinical and perioperative outcomes. Asian J Urol. 2022;9(2):119–24. 35509484 10.1016/j.ajur.2021.05.004 PMC9051357

[26] Subramanian VS, Nguyen CT, Stephenson AJ, Klein EA. Complications of open primary and post-chemotherapy retroperitoneal lymph node dissection for testicular cancer. Urol Oncol. 2010;28(5):504–9. 19097812 10.1016/j.urolonc.2008.10.026

[27] Parkin CJ, Sharma A, Winter M. How to do an open midline extraperitoneal bilateral retroperitoneal lymph node dissection for non-seminomatous germ cell tumour. ANZ J Surg. 2023;93(1–2):337–8. 36448646 10.1111/ans.18182

[28] Sheinfeld J, Feldman DR, DiNatale RG, Bosl GJ. Altering the natural history of surgical relapse in testicular cancer: suboptimal surgery and pneumoperitoneum. Eur Urol. 2019;76(5):612–4. 31327637 10.1016/j.eururo.2019.07.016

